# Airway Management in a Patient with Wolf-Hirschhorn Syndrome

**DOI:** 10.1155/2016/7070125

**Published:** 2016-09-26

**Authors:** John F. Gamble, Dinesh J. Kurian, Andrea G. Udani, Nathaniel H. Greene

**Affiliations:** Department of Anesthesiology, Duke University Medical Center, DUMC 3094, Durham, NC 27710, USA

## Abstract

We present a case of a 3-month-old female with Wolf-Hirschhorn syndrome (WHS) undergoing general anesthesia for laparoscopic gastrostomy tube placement with a focus on airway management. WHS is a rare 4p microdeletion syndrome resulting in multiple congenital abnormalities, including craniofacial deformities. Microcephaly, micrognathia, and glossoptosis are common features in WHS patients and risk factors for a pediatric airway that is potentially difficult to intubate. We discuss anesthesia strategies for airway preparation and management in a WHS patient requiring general anesthesia with endotracheal intubation.

## 1. Introduction

Wolf-Hirschhorn syndrome is a genetic disorder resulting from a microdeletion of the short arm of chromosome 4; the majority of these deletions arise as* de novo* events (85%), and the remainder as unbalanced translocations within the 4p16 chromosome [1]. WHS has a prevalence of 1 : 50,000–20,000 births, with a female to male ratio of 2 : 1 [1, 2]. Phenotypically, WHS presents as a constellation of characteristic features, including prenatal growth delay followed by short stature and slow weight gain, generalized hypotonia, variable degrees of intellectual disability, epilepsy, craniofacial dysgenesis, and various congenital midline fusion abnormalities [3, 4]. Cardiac lesions including septal defects, pulmonary stenosis, and patent ductus arteriosus are estimated to be present in approximately 50% of affected individuals [3]. WHS is associated with a high mortality rate, approximately 30% within the first two years of life, with the most common causes of death being lower respiratory tract infections and congenital heart disease/cardiac failure [1, 5]. We present the case of a 3-month-old female with WHS undergoing laparoscopic gastrostomy tube placement, with a focus on airway management in a pediatric patient with craniofacial abnormalities.

## 2. Case Presentation

**Figure 1:**
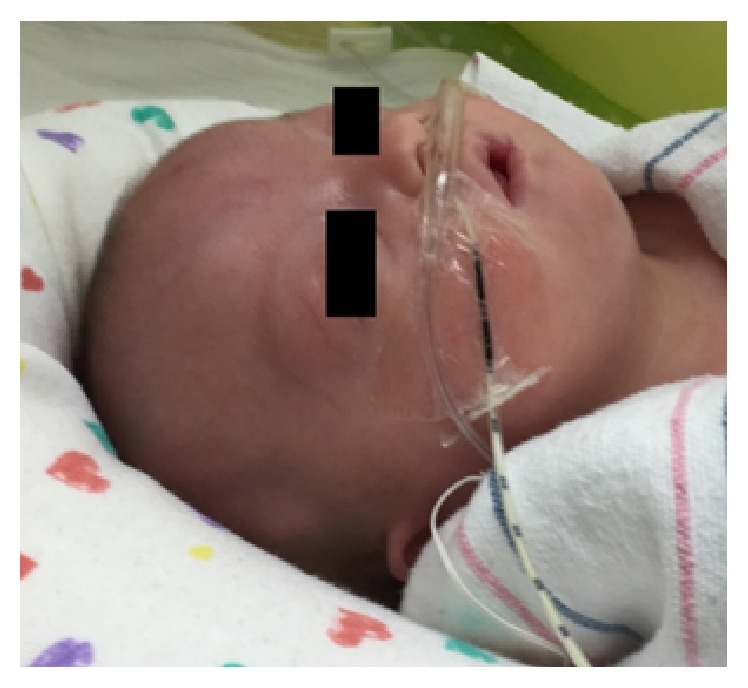
Preoperative image of the patient with Wolf-Hirschhorn syndrome, demonstrating microcephaly and micrognathia.

A 3-month-old female born with a prenatal diagnosis of Wolf-Hirschhorn syndrome was taken to the OR for planned laparoscopic gastrostomy tube placement due to poor oral feeding related to her muscular hypotonia. Her physical exam was remarkable for microcephaly with micrognathia and glossoptosis (Figure 1); a fiberoptic laryngoscopy exam performed by the otolaryngology service revealed moderate posterior tongue displacement, folded epiglottis, and arytenoid edema. She had no prior surgical history.

In the OR, standard monitors were placed, and the patient underwent spontaneous breathing induction with oxygen, nitrous oxide, and 4% sevoflurane. Her positioning was optimized with the use of a shoulder roll and gel head ring, and mask ventilation was performed without difficulty. Available at the bedside were a laryngoscope with MacIntosh and Miller blades, Glidescope, and fiberoptic bronchoscope. An initial intubation attempt was made via direct laryngoscopy using a Miller 1 blade, with anterior cricoid pressure applied by a second anesthesiologist. A grade 2 view was obtained and a 3.5 cuffed tube passed through the vocal chords without difficulty. Surgery was performed uneventfully, and the patient was transferred to the pediatric intensive care unit, intubated and in stable condition.

## 3. Discussion

Children with WHS are born with congenital abnormalities requiring special consideration when administering an anesthetic, particularly when it comes to airway management. Few cases discussing anesthetic management in patients with WHS have been identified. Previously reported approaches to airway management describe difficulty with intubation, attributed primarily to poor visualization of the glottis [5, 6]. In a case of a 33-month-old patient undergoing bilateral tympanoplasty and myringotomy, Choi et al. describe obtaining anterior, posterior, and lateral radiography of the cervical spine, in addition to performing a standard airway evaluation, to assist in identification of airway abnormalities [7]. Two cases describe the use of downsized endotracheal tubes, either as a result of difficulty with intubation or in an attempt to minimize the potential for airway challenges [5, 7]. None of the cases reviewed describes the use or availability of additional equipment or anesthesia providers to assist with airway management.

It was our experience that, with optimization of patient positioning and assistance of a second provider, successful tracheal intubation may be achievable via direct laryngoscopy. Additional airway adjuncts and means of indirect laryngoscopy, though important to have them readily available, may not always be required for successful management of a challenging pediatric airway.
